# Localization of post-disaster psychosocial care in China

**DOI:** 10.3402/ejpt.v5.26525

**Published:** 2014-12-09

**Authors:** Sujuan Zhang

**Affiliations:** National Disaster Reduction Center of Ministry of Civil Affairs of China, Beijing, People's Republic of China

**Keywords:** disaster relief, psychosocial care, spiritual pain, culturally sensitive, mental health policy, research

## Abstract

Disaster is not independent of society and culture and always happens in specific cultural and social contexts. Cultural and social characteristics influence the responses of people affected by disaster, as well as the process of disaster relief.

As one of the countries in the world that suffer most from natural disasters, various ethnic groups in China vary greatly in psychology and behavior characteristics after major disasters due to different geographical environments and economic and political conditions. To launch an effective post-disaster psychosocial care, 1) it is necessary to consider how to satisfy material, health, and other fundamental biological needs of affected people; 2) it is necessary to relieve disaster victims of their mental pain (spiritual in Chinese) and help them restore their psychological health; 3) it is necessary to revitalize the seriously unbalanced communities affected by disasters so that these communities would burst with vitality again. In addition, it is necessary to take specific ethnic and regional culture into account when helping people in these areas gradually achieve social adaptation and cultural identification. All these require us to intensify our efforts in the following four aspects: 1) to strengthen legislation and institutional construction in this field; 2) to help citizens master the most fundamental psychological principles and methods of coping with disasters to enable timely self-aid and mutual-aid; 3) to build a national database of the post-disaster psychosocial care teams; 4) to continue the research on disaster psychology, so as to provide a scientific basis as well as techniques and methods for implementing disaster relief efforts in a scientific way.

